# Can type of school be used as an alternative indicator of socioeconomic status in dental caries studies? A cross-sectional study

**DOI:** 10.1186/1471-2288-11-37

**Published:** 2011-04-02

**Authors:** Chaiana Piovesan, Monica Carneiro Pádua, Thiago Machado Ardenghi, Fausto Medeiros Mendes, Gabriela Cunha Bonini

**Affiliations:** 1 Department of Orthodontics and Pediatric Dentistry, School of Dentistry, University of São Paulo, São Paulo, Brazil; 2 School and Center for Dental Research, São Leopoldo Mandic, Campinas, São Paulo, Brazil; 3 Department of Stomatology, University of Santa Maria, Rio Grande do Sul, Brazil

## Abstract

**Background:**

Despite the importance of collecting individual data of socioeconomic status (SES) in epidemiological oral health surveys with children, this procedure relies on the parents as respondents. Therefore, type of school (public or private schools) could be used as an alternative indicator of SES, instead of collecting data individually. The aim of this study was to evaluate the use of the variable type of school as an indicator of socioeconomic status as a substitute of individual data in an epidemiological survey about dental caries in Brazilian preschool children.

**Methods:**

This study followed a cross-sectional design, with a random sample of 411 preschool children aged 1 to 5 years, representative of Catalão, Brazil. A calibrated examiner evaluated the prevalence of dental caries and parents or guardians provided information about several individual socioeconomic indicators by means of a semi-structured questionnaire. A multilevel approach was used to investigate the association among individual socioeconomic variables, as well as the type of school, and the outcome.

**Results:**

When all significant variables in the univariate analysis were used in the multiple model, only mother's schooling and household income (individual socioeconomic variables) presented significant associations with presence of dental caries, and the type of school was not significantly associated. However, when the type of school was used alone, children of public school presented significantly higher prevalence of dental caries than those enrolled in private schools.

**Conclusions:**

The type of school used as an alternative indicator for socioeconomic status is a feasible predictor for caries experience in epidemiological dental caries studies involving preschool children in Brazilian context.

## Background

Significant decline of caries prevalence has been observed in developed and developing countries in the last decades [1-3]. This impressive reduction of caries indices is attributed to the use of different fluoride-releasing vehicles such as fluoride toothpastes and the Ӿuoridation of tap water and changes in the pattern and amount of extrinsic sugar consumption [4,5].

This overall improvement was concurrent with an increasingly unequal distribution of the disease, with higher caries rates affecting deprived areas [6]. The association between socioeconomic factors and prevalence of dental caries in preschool children has been consistently demonstrated [7,8]. Nevertheless, most of the studies have used individual socioeconomic data [6,9-11]. Despite the importance of collecting individual data of socioeconomic status (SES) in epidemiological oral health surveys with children, this procedure relies on the parents as respondents [11,12]. The collection of variable 'type of school' as indicator of SES could be obtained more easily, decreasing the duration and the cost of the study. In fact, a high level of disease is observed in children enrolled in public school [13-15].

However, there are no previous studies comparing the strength of the association between the presence of dental caries and the type of school (public or private schools) used as an alternative indicator of SES, instead of collecting data individually. Therefore, this study aimed to evaluate the use of the variable type of school as an indicator of SES instead of individual data in an epidemiological survey about dental caries in Brazilian preschool children.

## Methods

### Sample

A cross-sectional study was conducted in a representative sample of 1 to 5 years-old preschool children from Catalão-GO, Brazil. Catalão is a Brazilian municipality with 79.618 inhabitants, located at the south of Goiás state. The infant mortality is 12.58 per 1000 live births and Human Development Index is 0.818 (slightly higher than the Brazilian average of 0.792). The city has added 0.7 mg/L F- to its controlled water supply since 1991.

Fifteen schools, twelve privates and three publics, were selected for the study. Firstly, three public schools were selected. These schools were selected because they represent the major schools in the city, corresponding to nearly 85% of the children attending and they are urban schools. Secondly, four private schools were randomly selected according to the location of each public school. This procedure permitted to select children living in similar areas and a comparable number of children in private and public schools. A total of 2400 children were enrolled in these schools in 2009.

For the sample size calculation, we adopted a standard error of 5%, a confidence interval level of 95% and an expected prevalence of 50%. In addition, 20% to non-response were applied. The minimum sample size to satisfy the requirements was estimated to be 400 children. The decision to use a prevalence of 50% was due to lack of information of the actual prevalence of the outcome in city.

### Data collection

Data were collected through clinical oral examinations and structured questionnaire. One examiner and one support member participated in the study. The examiner was previously trained for data collection before the survey. Theoretical, clinical training and calibration exercises were arranged for a total of 36 hours. During the calibration process, the examiner re-examined approximately 10% of the sample (45 children), distributed throughout all 15 schools, to assess intra-examiner reliability.

Dental examination used international criteria standardized by the World Health Organization for oral health surveys [16]. Children were examined in a room with natural light, using CPI probes and plane dental mirrors. The clinical examination recorded the presence of dental caries lesions at surface level.

Socioeconomic characteristics were informed by parents or guardians. The questionnaire presented a series of questions regarding socioeconomic and demographic characteristics such as age and gender of child, household overcrowding, house ownership, if the child resides with mother and father, parents' educational level and household income. Household overcrowding refers to the average number of dwellers per bedroom in households. Educational level compared fathers and mothers who completed 8 years of formal instruction, which in Brazil corresponds to primary school, with those who did not. Household income was measured in terms of the Brazilian minimum wage (BMW), a standard for this type of assessment, which nearly corresponded to 280 US dollars during the period of data gathering. The feasibility of the questionnaire was previously assessed in a sample of 20 parents during the calibration process.

Furthermore, the type of school of the child was recorded, classifying as public or private school.

### Statistical analysis

Since children are clustered in the type of school, a multilevel approach was used to investigate the association among explanatory variables and the outcome. MLwin software (MLwiN 2.10, Centre for Multilevel Modeling, Bristol, UK) was used to perform the analyses.

Then, the explanatory variables were divided in two levels:

- First level - child: gender (male or female); age (< 4 years or ≥ 4 years); resides with mother and father (no or yes), household overcrowding (≤ 0.5, 0.5 - 0.79 or ≥ 0.8 person/room), house ownership (no or yes), parents' level of education (up to 8 yrs or more than 8 years) and household income (up to 5 BMW or more than 5BMW).

- Second level - type of school (private or public).

Univariate analyses were initially performed and the unadjusted odds ratio (OR) and 95% confidence interval (95%CI) were calculated. Subsequently, multilevel logistic regression was done using a forward stepwise approach. Initially, in the first model, we considered a level of 0.20 for entry into the model and a level of 0.05 to retain the variable in the model. We also checked the possible interactions present in all models.

After that, we performed an alternative model using the type of school instead of individual SES indicators. In order to check the strength of association of the outcome with individual SES or type of school data, we made another final model introducing the individual variables considering a level of 0.20 in the unadjusted analysis and type of school, keeping in the final model all variables regardless of the final level of significance. We calculated the adjusted OR for the variables present in the final models and the variance of each final model.

In order to confirm if the type of school can be a proxy measure for individual data about SES, chi-square tests were applied to assess the association between individual socioeconomic indicators and type of school among the same subjects.

### Ethics

This study observed international statutes and national legislation on ethics in research involving human beings. All parents or guardians signed a term of consent. The study protocol was approved by the Committee of Ethics in Research of Dental School and Center for Dental Research, São Leopoldo Mandic.

## Results

The response rate was 100% of all children invited. A total of 411 children, 50.4% boys and 49.6% girls, were recruited for the study. Intra-examiner agreement for dental caries was 0.9.

Table 1 summarizes the demographic characteristics of the sample. Prevalence of dental caries was 34.3%. The percentage of children participating was similar in different type of schools. Most children were older than 4 years-old and resided with mother and father; their parents mostly had complete primary education and more than a half of the families earned less than five BMW. The majority of the sample had an average number of dwellers per bedroom in households of 0.8.

**Table 1:** Unadjusted assessment of socioeconomic variables associating with prevalence of dental caries

	Without caries	With caries	Odds Ratio (95% CI)	p*
**Level 2: school (n = 411 children in 15 schools)**				

**Type of school**				
Private	150 (73.2)	55 (26.8)		
Public	120 (58.2)	86 (41.7)	1.99 (1.20 to 3.29)	0.008

**Level 1: children (n = 411)**				

**Gender**				
Male	132 (63.8)	75 (36.2)		
Female	138 (67.6)	66 (32.3)	0.84 (0.55 to 1.26)	0.394
**Age**				
< 4 yrs-old	65 (60.7)	42 (39.2)		
≥ 4 yrs	205 (67.4)	99 (32.6)	0.82 (0.51 to 1.32)	0.414
**Resides with mother and father?**				
Yes	205 (65.3)	109 (34.7)		
No	65 (67.0)	32 (33.0)	0.91 (0.56 to 1.50)	0.721
**Household overcrowding (persons/room)**				
≤ 0.5	124 (77.0)	37 (23.0)		
0.5 - 0.79	104 (70.3)	44 (29.7)	1.39 (0.82 to 2.34)	0.120
≥ 0.8	42 (41.2)	60 (58.9)	4.76 (2.70 to 8.41)	< 0.001
**House ownership**				
No	77 (67.0)	38 (33.3)		
Yes	193 (65.2)	103 (34.8)	1.13 (0.71 to 1.80)	0.607
**Mother's level of education**				
Up to 8 yrs	55 (45.4)	66 (54.5)		
More than 8 yrs	213 (74.0)	75 (26.0)	0.30 (0.19 to 0.47)	< 0.001
**Father's level of education**				
Up to 8 yrs	55 (47.4)	61 (52.6)		
More than 8 yrs	203 (75.0)	68 (26.0)	0.31 (0.19 to 0.48)	< 0.001
**Household income**				
Up to 5 BMW	146 (57.2)	109 (42.7)		
More than 5 BMW	124 (79.5)	32 (20.5)	0.35 (0.22 to 0.57)	< 0.001

95% CI = 95% confidence interval. BMW = Brazilian Minimum wage (about US$280.00/month during the period of data gathering) * significance evaluated by Wald test

At the child level (first level) household overcrowding, parents' education and household income showed significant association in the unadjusted analysis. Type of school (second level) was also significantly associated with presence of dental caries (Table 1).

The first multiple multilevel analysis revealed that children whose mothers have not completed primary education and those with lower household income had higher caries experience than other children (Table 2, model 1). When type of school was put in this model, this variable lost the significance and was removed of the first final model. Other variables which presented significant association in the univariate analysis were also tested but they were not significant after the adjustments (Table 2, model 1).

**Table 2:** Multiple logistic multilevel analysis of association among independent variables and presence of dental caries

	Model 1		Model 2		Model 3	
	**Odds ratio (95%CI)**	**p**	**OR (95%CI)**	**p**	**OR (95%CI)**	**p**

**Type of school**						

Private						
Public			1.99 (1.20 - 3.29)	0.008	0.87 (0.48 -1.57)	0.647

**Mother's level of education**						

Up to 8 yrs						
More than 8 yrs	0.39 (0.24 - 0.63)	< 0.001			0.38 (0.26 - 0.63)	< 0.001
**Household income**						
Up to 5 BMW						
More than 5 BMW	0.49 (0.29 -0.82)	0.007			0.46 (0.26 - 0.82)	0.007

**p (entire model)**		< 0.001		0.008		<0.001

**Variance (standard error)**	0.029 (0.068)		0.055 (0.077)		0.036 (0.071)	

95% CI = 95% confidence interval

In the second final model, we tested the use of type of school without the individual SES variables, and we could observe that the child enrolled in a public school had twice as much chance of presenting with dental caries than a child attended in a private school (table 2, model 2).

When we put significant individual variables with type of school, we could observe that type of school was not significant due to the collinearity of these variables. This final model with both individual SES data and type of school is demonstrated in the table 2 (model 3). The model 2 presented higher variance than the other models (table 2).

Table 3 expressed the association between individual socioeconomic indicators of children and type of school. Positive associations were found between mother's level of education, household income and household overcrowding with type of school that the children were attended.

**Table 3:** Association between individual socioeconomic indicators of children and type of school

	Private	Public	p*
**Resides with mother and father?**			
Yes	163 (51.9)	151 (48.1)	
No	42 (43.3)	55 (56.7)	0.172
**Household overcrowding (persons/room**)			
≤ 0.5	127 (78.9)	34 (21.1)	
0.5 - 0.79	66 (44.6)	82 (55.4)	
≥ 0.8	12 (11.8)	90 (88.2)	< 0.001
**House ownership**			
Yes	52 (45.2)	63 (54.8)	
No	153 (51.7)	143 (48.3)	0.286
**Mother's level of education**			
Up to 8 yrs	18	103	
More than 8 yrs	185	103	< 0.001
**Father's level of education**			
Up to 8 yrs	31 (26.7)	85 (73.3)	
More than 8 yrs	164 (60.5)	107 (39.5)	< 0.001
**Household income**			
Up to 5 BMW	73 (28.6)	182 (71.4)	
More than 5 BMW	132 (84.6)	24 (15.4)	< 0.001

* calculated by chi-square test.

## Discussion

The present study showed that the type of school, used as an alternative indicator for SES, is a feasible variable for the prevalence of dental caries; this is the most important result of the current study. Actually, previous studies have shown that children from public schools have higher prevalence of dental caries [13,17]. However, to the best of our knowledge, no earlier study compared the possibility of using type of school instead of individual socioeconomic indicators, and the implications of this procedure in Brazilian context. This is important from scientific perspective mainly because it may contribute to the study epidemiological patterns where the gathering of individualized socioeconomic information is not feasible.

In our study, socioeconomic backgrounds were assessed by means of different type of variables. The collection of subject-level data about income, educational levels and household crowding requires the application of an inquiry to the children's parents and these would require additional efforts. Therefore, the use of type of school can facilitate epidemiological surveys of oral health by simplifying the data collection. Although household income and mother's level of education remained associated with the outcome after the adjustment, and the type of school lost the significance in Model 3, the type of school proved to be a good predictor when used alone (Model 2).

Thus, the variable type of school was sensitive for discriminating different oral health conditions. Children from higher socioeconomic backgrounds generally were enrolled in private school as opposed to children from lower SES backgrounds who attended mainly in public school. Previously, associations were observed among individual SES variables and type of school [17,18]. This finding was also observed in our study.

On the other hand, the use of type of school should be considered with caution. Firstly, the association with presence of dental caries was stronger when the individual data were considered. We could observe this fact in the final model 3 since the type of school was not significantly associated after adjustment by the individual indicators. Moreover, the final model using type of school presented higher variance, and therefore, studies using this variable as alternative of SES will require a greater sample size than the data collected individually.

When we considered the individual effect of socioeconomic status on caries prevalence, we found a significant relation between household income and mother's schooling with the outcome. Children whose mothers had not completed primary education and those with lower household income had higher caries experience than their counterparts.

Theoretical explanation on the link between socioeconomic status and oral health focuses on the effect of material deprivation on individual lifestyle decisions [19]. Socioeconomic factors can interact with social characteristics to produce different health effects across groups. Concerning mother's schooling and household income, low educational level may leads to reduced income, unemployment and poor occupational status; these conditions influence health behaviors [20]. Risk behaviors lie in the causal pathway between socioeconomic position and oral health and are more prevalent among socioeconomically disadvantaged groups [21].

Concerning to the type of schools, the high level of dental caries found among preschool children attending public schools in the current study represents a major dental public health problem in Brazil. In general, higher levels of dental decay are found in areas of social deprivation, for example, public schools [4,13,14]. Probably, this may be due to the environment in which the child is inserted. Oral health is determined by a variety of activities associated with relationships, self-esteem and opportunities to make healthier decisions. Therefore, schools could be considered appropriate settings for health promotion for children, since the school may provide an environment for improving health, self-esteem, behaviors and life skills [22,23].

This study has some limitations and results should be interpreted with caution. We followed a cross-sectional design, and therefore, it is not possible to establish a temporal relationship. It is important that the use of type of school as an alternative indicator of individual SES in epidemiological dental caries studies be confirmed longitudinally, where the time when the child is inserted in school can be monitored.

## Conclusion

The type of school could be used as an alternative measure of socioeconomic status in epidemiological dental caries surveys in the Brazilian context when is not possible to collecting data individually.

## Competing interests

The authors declare that they have no competing interests.

## Authors' contributions

All authors read and approved the final manuscript. CP wrote the manuscript and collaborated in the statistical analysis. MCP collected the data and wrote the paper. TMA collaborated in the statistical analysis and revised the manuscript. FMM performed the statistical analysis, have been involving in writing the manuscript and revised the manuscript. GCB coordinated the study and wrote the manuscript.

## Pre-publication history

The pre-publication history for this paper can be accessed here:

http://www.biomedcentral.com/1471-2288/11/37/prepub
